# RRG-GAN Restoring Network for Simple Lens Imaging System

**DOI:** 10.3390/s21103317

**Published:** 2021-05-11

**Authors:** Xiaotian Wu, Jiongcheng Li, Guanxing Zhou, Bo Lü, Qingqing Li, Hang Yang

**Affiliations:** 1 College of Opto-Electronic Engineering, Changchun University of Science and Technology, Changchun 130022, China; wuxiaotian@ciomp.ac.cn; 2 Changchun Institute of Optics, Fine Mechanics and Physics, Chinese Academy of Sciences, Changchun 130033, China; lvbo@ciomp.ac.cn (B.L.); liqingqing17@mails.ucas.ac.cn (Q.L.); 3 School of Informatics, Xiamen University, Xiamen 361005, China; 23320201153997@stu.xmu.edu.cn (J.L.); joaquinchou@stu.xmu.edu.cn (G.Z.)

**Keywords:** computational imaging, deep learning, image restoring, non-uniform deblurring

## Abstract

The simple lens computational imaging method provides an alternative way to achieve high-quality photography. It simplifies the design of the optical-front-end to a single-convex-lens and delivers the correction of optical aberration to a dedicated computational restoring algorithm. Traditional single-convex-lens image restoration is based on optimization theory, which has some shortcomings in efficiency and efficacy. In this paper, we propose a novel Recursive Residual Groups network under Generative Adversarial Network framework (RRG-GAN) to generate a clear image from the aberrations-degraded blurry image. The RRG-GAN network includes dual attention module, selective kernel network module, and residual resizing module to make it more suitable for the non-uniform deblurring task. To validate the evaluation algorithm, we collect sharp/aberration-degraded datasets by CODE V simulation. To test the practical application performance, we built a display-capture lab setup and reconstruct a manual registering dataset. Relevant experimental comparisons and actual tests verify the effectiveness of our proposed method.

## 1. Introduction

Computational imaging is a new interdisciplinary subject in recent years, which offers imaging functionalities and convenient design beyond traditional imaging design [1]. It emphasizes the task-oriented global optimization design in the full imaging chain, and systematically balances the system resource dependence in the physical and computing domain. Computational imaging has many sub-topics according to different imaging backgrounds [2,3]. This paper will focus on the simple lens imaging system and its restoring method.

**Figure 1:**
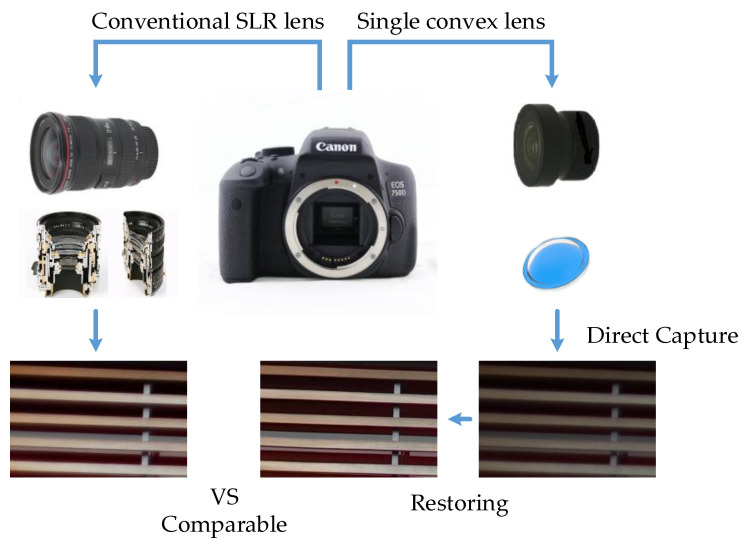
The schematic diagram of single-convex-lens computational imaging method.

Aberration is not only the main consideration in the optical design stage, but also a factor limiting the imaging quality in the actual use due to the change of aperture, object distance, and other factors. To minimize optical aberrations, the manufacturing of photographic lenses has become increasingly complex. Optical designers systematically balance optical aberrations and design constraints (such as focal length, field of view, and distortion). They utilize a combination of several lens elements with various materials and shapes to achieve a close-to-perfect optical design, which will result in a significant impact on the cost, size, and weight. The simple lens computational imaging method provides an alternative way to achieve high-quality photography. It simplifies the design of the optical-front-end to a single-convex-lens, and delivers the correction of optical aberration to a dedicated computational restoring algorithm, as shown in Figure 1. As aberration is a common problem in many optical imaging systems, aberration correction algorithm will have great significance to improve the quality of other optical imaging systems, and has broad application prospects.

In this paper, we will build a single-convex-lens imaging system, and further study the restoring method of aberration degraded image. The contributions of this paper are as follows:

1. We collect sharp/aberration-degraded datasets by CODE V (https://www.synopsys.com/optical-solutions/codev.html, accessed on 11 May 2021) simulation and manual registering, which will be publicly released on Github for further researches (https://github.com/wuzeping1893/RRG-GAN-single-convex-lens, accessed on 11 May 2021). To the best of our knowledge, this is the first dataset for the single-convex-lens computational imaging field.
2. We propose the application of deep-learning-only-based methods for image denoising and deblurring to the special case of single-lens camera images restoring, in contrast with optimization-based methods with great improvement in efficiency and efficacy.
3. By redesigning the generator network, the proposed RRG-GAN network includes the dual attention module, selective kernel network module, and residual resizing module. It has better multi-scale feature extraction and fusion ability, which makes the network have better recovery effect.

The following sections are arranged as follows: Section 2 briefly describes the related work and the chosen direction of this paper. Section 3 introduces hardware implementation of single-convex-lens imaging system and our proposed RRG-GAN restoring network. Section 4 provides the experimental preparation, results, and analysis. Section 5 discusses our work and future research. Section 6 presents the conclusions of our methods.

## 2. Related Work

### 2.1. Single-Lens Imaging System

The idea of single-convex-lens computational imaging was first proposed by Schuler et al. [4]. They utilized a single-convex-lens as the only optical element, measured the non-uniform point spread function (PSF) grid covering via an automated calibration procedure, and eliminated the effect of aberration through a non-stationary convolution [4]. Following Schuler’s work, Felix improved the restoring method by the proposed cross-channel prior [5].

In order to expand the field of view (FOV), Peng [6] proposed a thin-plate optics computational imaging method using a single-Fresnel-lens, another construction method of single lens. Similar to the single-convex-lens computational imaging method, some scholars proposed dedicated image restoring methods for computational imaging systems using diffractive optical elements (DOEs) [7,8].

### 2.2. Motion Deblurring Algorithms

Most computational imaging methods are all indirect imaging, which means the source image data obtained from the sensor is seriously affected by the aberration. Therefore, the design of robust computational restoring algorithms is the key issue of these computational imaging systems. Usually, the aberration correcting and restoring methods refer to some ideas of motion deblurring algorithms. The motion deblurring algorithms are mainly based on two kinds of theories: one is traditional optimization, the other is deep learning.

The motion deblurring methods based on the optimization framework belong to a theoretical method driven by the physical model. Image deblurring is a typical ill-posed problem, the restoration methods usually need to add prior constraints and integrate them into optimization framework to make ill-conditioned problems solvable. Representative image priors mainly include L0 gradient [9], gradient sparsity [10,11], dark channel [12], color-line [13] etc. The optimization framework adopted mainly includes maximum a posteriori approach (MAP) and variational expectation maximization (VEM).

The motion deblurring methods based on deep learning belong to a data-driven theoretical implementation method, but the pointcuts and data-driven implementation methods are different. Chakrabarti [14] used a deep learning network to estimate the blur kernel Fourier coefficient. Wang trained a discriminative classifier to distinguish blurry and sharp images as the regularization term in the maximum a posteriori (MAP) framework [15]. Similarly, based on the MAP framework, Ren et al. [16] further obtained the image priors and blur kernel priors all by deep learning network, and thus improved the image restoration effect in the case of complex or large-scale blur kernel. The above methods are mainly based on the combination of deep learning and traditional optimization: the traditional optimization method is responsible for the main process, while the deep learning algorithm is used to improve the robustness of various priors. Due to the joint application of deep learning and optimization theory, these methods improve the recovery effect, but there is no improvement in efficiency.

The end-to-end deep network is another way to implement computational recovery. Nah et al. [17] proposed an end-to-end multi-scale convolutional neural network to realize the deblurring algorithm. Zhang et al. [18] make use of the feature extraction advantage of RNN and the weight learning advantage of CNN to realize a non-uniform deblurring neural network. Zhou et al. [19] proposed a multi-stream bottom-top-bottom attention network, which can effectively facilitate feature extracting and reduce computational complexity. With the development of generative adversarial networks (GAN), the implementation method of directly generating end-to-end restoration images and ignoring the physical model process is also applied in the field of image deblurring, which is represented as Deblur-GAN, proposed by Kupyn [20].

### 2.3. Motivation of This Paper

The motivation of this paper is to utilize the deep learning method instead of the previous optimization-based methods [4,5] to improve the efficacy and efficiency.

From the perspective of implementation effect, previous methods will fail when the size of blurry kernel is large. In contrast, our experiments in Section 4 will prove that the deep learning restoration method has better robustness and better subjective restoration effect in this aspect.

In terms of operational efficiency, the optimization methods usually require several iterations, which means they are difficult to apply in practical engineering. In contrast, the deep learning end-to-end restoring methods are more efficient, although they require additional hardware resources such as GPU.

We make appropriate improvements on the Deblur-GAN [20] restoring method, which is originally used in motion blur recovery. The improvements mainly reflect the restructuring of the generator network, which includes the dual attention module, selective kernel network module, and residual resizing module. These improvements mean the network restoration has better multi-scale feature extraction and fusion ability, as shown in Section 4.

## 3. Methodology

### 3.1. Hardware Implementation of Single-Convex-Lens Imaging System

#### 3.1.1. Self-Made Optical Lens

**Figure 2:**
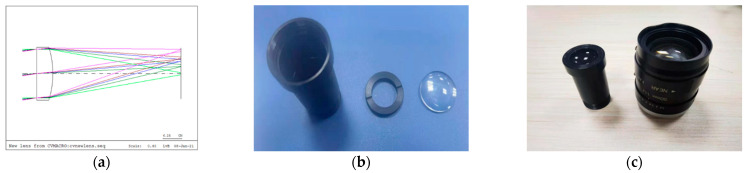
The procedure of self-made optical lens: (**a**) The light path of lens using CODE V software; (**b**) The components of self-made optical lens; (**c**) The assemble of self-made optical lens and its corresponding standard SLR lens.

The self-made optical lens consists of a flat-convex lens, a gland ring, and a lens barrel. The flat-convex lens is the only optical element to converge light. The focal length of the lens is 50 mm with 15° field-of-view (FOV), which is larger than the previous work [4,5]. The gland ring is used to fix the lens and act as a simple aperture. Our self-made optical lens has a corresponding standard single-convex-lens reflex (SLR) lens with the same focal length and field angle, as shown in Figure 2.

The parameters of our self-made optical lens can be found in CODE V source project documents, which is available on Github project page. The light path analysis in Figure 2a and the subsequent simulations are all based on the premise of infinite object distance. For the case of small object distance, the change of PSF is varied very sharply. We do not discuss these situations in this paper.

#### 3.1.2. Aberration Analysis and Imaging Model

The aberrations produced by the single-convex-lens will lead to the degradation of image quality. These aberrations can be divided into on-axis aberrations and off-axis aberrations. The on-axis aberrations mainly include the longitudinal chromatic aberration and the axial spherical aberration. The off-axis aberrations mainly include lateral chromatic aberration, coma, curvature of field, astigmatism, and distortion. The overall influence of the above aberrations on imaging is reflected in the complexity of the point spread function (PSF). The PSFs are different in the RGB channels, global non-uniform with large kernel size.

Affected by optical aberrations, the non-uniform blurry imaging model in RGB channel is described in Equation (1):

(1) $$O_{c}(i,j)=\sum_{\left(m,n\right)}I_{c}(m,n)K_{c}(m,n,i,j)+N_{c}(i,j),c\in \{R,G,B\}$$

In Equation (1), $O_{c}(i,j)$ denotes the source image collected by the imaging system, (i,j) depicts the pixel position of the observed image space; $I_{c}(m,n)$ represents the ground truth image, (m,n) is the pixel position of the ground truth image space; $K_{c}(m,n,i,j)$ describes the non-uniform point spread function (PSF) of the motion blur; $N_{c}(i,j)$ is the CMOS noise. The observed blurry image $O_{c}(i,j)$ is known, the ground truth image $I_{c}(m,n)$, the point spread function $K_{c}(m,n,i,j)$, and the CMOS noise $N_{c}(i,j)$ are all unknown, which means it is a typical ill-posed problem in mathematics.

Mathematically, the ill-posed problems can be solved by adding prior constraints under the optimization framework. However, the optimization-based restoring methods usually require multiple iterations. The computing time of a single image with 1920 × 1080 resolution is usually more than 5 min, which is unacceptable in industrial applications. With the continuous development of deep learning theory and GPU acceleration hardware technology, the restoring methods based on deep learning show great advantages in effect and performance [21,22]. The restoring method proposed in this paper is based on the deep learning theory and realizes the image restoration procedure in an end-to-end way.

### 3.2. Proposed Restoring Method

#### 3.2.1. Network Architecture

**Figure 3:**
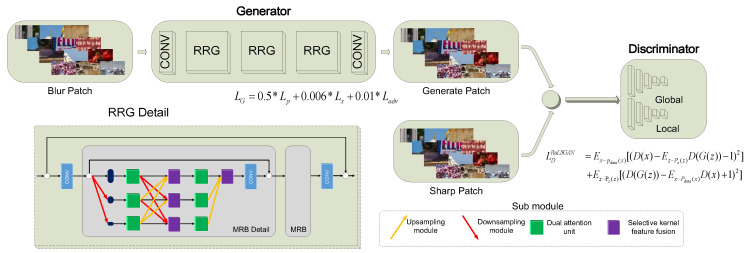
The RRG-GAN network architecture. (* denotes the multiplication operation).

We propose a novel generative adversarial network (GAN) to generate a clear image from the aberrations-degraded blurry image. The generative adversarial network (GAN), proposed by Goodfellow [23], became one of the most attractive schemes in deep learning in recent years. GAN utilize a discriminator network as a special robust loss function instead of the traditional hand-crafted loss function, which improves the performance and robustness of deep networks [24,25]. Specifically, we adopt the recursive residual groups (RRG) network [26] as the generative model G, thus our proposed generative adversarial network is named as RRG-GAN. The discriminator of RRG-GAN is a double scale discriminator network [20]. The proposed network architecture is shown in Figure 3.

#### 3.2.2. Discriminator Network: Double-Scale Discriminator Network

The discriminator of RRG-GAN is a double-scale discriminator network. The original GAN network’s discriminator maps the entire input to a probability of judging whether the input sample is a real image [20]. However, this method does not work very well for high-resolution and high-definition detailed images. PatchGAN [27] maps the entire input to an n*n patch matrix to classify, and then averages them to obtain the final output of the discriminator. Therefore, PatchGAN can make the model pay more attention to the details of the local image to achieve better results. The double scale discriminator, proposed by Kupyn [20], further improves PatchGAN by adding a global image view. The combine use of local and global view was proved more suitable for deblurring task [20].

The loss function of discriminant network is RaGAN-LS loss, also proposed by Kupyn [20], as shown in Equation (2), which can be seen as an upgrade version of LSGAN loss function [28].

(2) $$\begin{array}{cc} L_{D}^{RaLSGAN} & =E_{x\sim p_{data}(x)}[{\left(D(x)-E_{z\sim P_{z}(z)}D(G(z))-1\right)}^{2}]\\ & +E_{z{\sim P}_{z}(z)}[{\left(D(G(z))-E_{x\sim P_{data}(x)}D(x)+1\right)}^{2}] \end{array}$$

where x and z respectively represent the real image and generator G’s latent variables input. $P_{z}(z)$ is the probability distribution of z, $P_{data}(x)$ is the probability distribution of the dataset. G(z) denotes the generator of z. D(x) represents real image x’s double-scale discriminator. D(G(z)) represents G(z)’s double-scale discriminator.

#### 3.2.3. Generator Network: RRG-Net

The generator network is implemented by cascading three recursive residual groups (RRGs) as shown in Figure 3. RRG is proposed in the structure of Cycle ISP network by Google Research Institute [26]. Cycle ISP network is an end-to-end network, which can simulate the real CMOS noise degradation, and then achieve image restoration, it can realize bidirectional conversion and circulation in sRGB domain and RAW domain. In this work, there is no cycle in the construction of generation network, we only use the one-way generation function in Cycle ISP network. The cascading recursive residual groups can be described as:

(3) $$T_{0}=M_{s}(I_{in}),T_{d}=RRG_{N}(\ldots RRG_{1}(T_{0})),I_{out}=M_{E}(T_{d})$$

where $M_{s}$ is the starting convolution operation on the input color image $I_{in}\in R^{H\times W\times 3}$ to get the low-level feature parameters $T_{0}\in R^{H\times W\times C}$; the high-level features of $T_{d}\in R^{H\times W\times C}$ are obtained by the iteration of recursive residual groups (RRGs); then $T_{d}$ is performed the final convolution operation $M_{E}$ on the high-level features $T_{d}$ is to obtain the reconstructed image $I_{out}\in R^{H\times W\times 3}$.

RRG’s framework is basically similar to the earlier proposed recursive residual network [29], but the construction of “group network” is more complex. In this paper, we choose multi-scale residual block (MRB) as the implementation of “group network”. The multi-scale residual block network contains several popular sub-modules to improve the performance of feature extraction [30]. These sub-modules include dual attention blocks [31], selective kernel networks [32], and residual resizing modules [33]. The dual attention blocks are used in each scale, which can simulate the process of human eyes searching for important information in the visual scene for centralized analysis while ignoring irrelevant information in the scene. The selective kernel networks are used in multi-scale. They can effectively learn to fuse important features extracted by the dual attention blocks. The residual resizing networks provide additional network parameters for learning in the inter-scale up-down-sampling operation, so as to further improve the performance of the whole network.

We use a hybrid three term loss function as the overall loss function for generator network, which is proposed in DeblurGAN-v2 [20]. The loss function is shown in Equation (4).

(4) $$L_{G}=0.5*L_{p}+0.006*L_{x}+0.01*L_{adv}$$

where $L_{p}$ is the similarity between two images, we use the SSIM (Structural Similarity) [34] measurement; $L_{x}$ denotes the perceptual loss, which computes the Euclidean loss on the VGG19 [35] feature maps to make the network pay more attention to learning coding perception and high-level semantic information; $L_{adv}$ represents the adversarial loss, which contains both global and local discriminator losses (for more details in Equation (4), please refer to Kupyn’s paper [20]).

## 4. Experiments and Results

### 4.1. Preparation of Dataset Based on CODE V Simulation Datasets

The optical aberrations are not the only factor of actual image degradation, which we will discuss later in the paper. The construction of simulation dataset based on CODE V software (Synopsys Corporate Headquarters, 690 East Middlefield Road Mountain View, CA, USA) is of great significance, which can provide reference for the evaluation of recovery algorithm, especially for the supervised learning method.

CODE V software, a famous optical design and analysis tool, has the excellent simulation function for optical aberration. We build a dataset based on the simulation function of CODE V. All the data sets used in this paper are from MIT Adobe FiveK dataset, which is mainly used in the research of tone adjustment of deep learning method [36].

The dataset consists of raw camera data and five groups of processed data tuned by professionals using Adobe professional image processing software. The dataset basically covers the natural scene, artificial scene, character photography, and other areas. This work selects a group of optimized data as the original verification data of the system.

**Figure 4:**
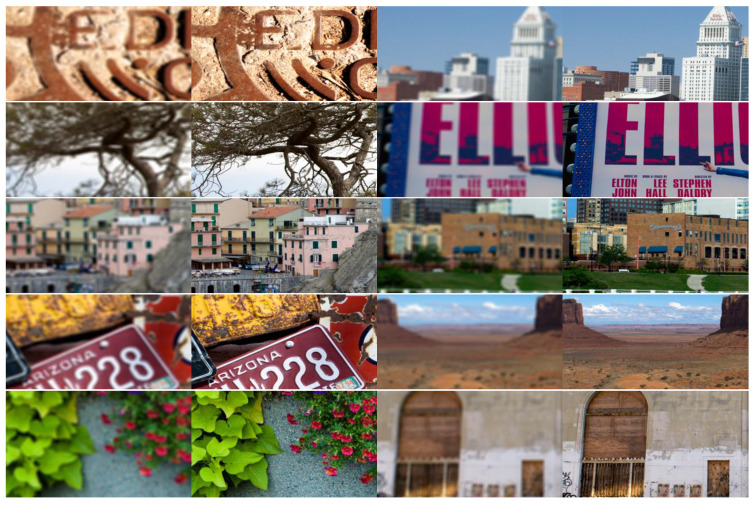
Part of data set based on CODE V simulation.

We manually filter out the experimental pictures which show obvious defocusing images, and finally select 200 experimental pictures including characters, text and natural scenes. Using the two-dimensional image simulation function of CODE V, we take the clear images as inputs, and obtain the simulation degraded images. Part of the simulation dataset is shown in the Figure 4.

In this way, we obtain 200 groups of supervised samples with true value and optical degradation simulation data. Overall, 128 groups are selected as training dataset, 36 groups as validation dataset and 36 groups as test dataset.

In the network training, we use the online random block strategy for all the comparison methods (except the optimization-based method). When we read the training samples, we randomly crop 256 × 256 blocks in the original image during the training. The epoch is set to 1000 rounds, that is, 128,000 training blocks are used in the network training process. The batch size is set to 8.

### 4.2. Algorithm Evaluation Based on CODE V Simulation Dataset

**Figure 5:**
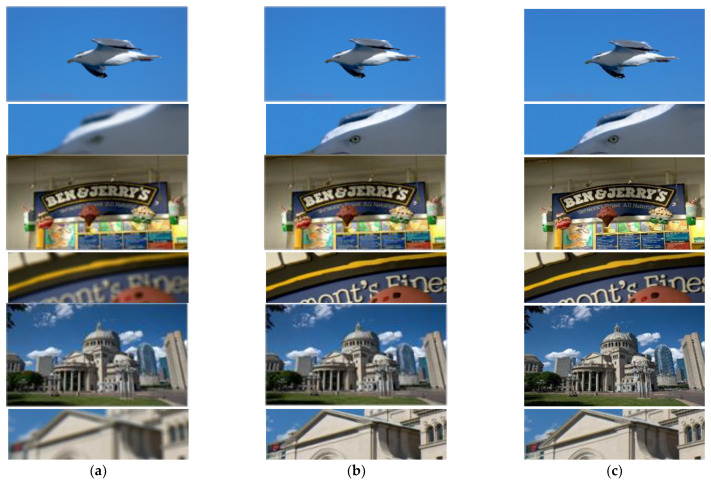
RRG-GAN restoring results based on CODE V simulation dataset: (**a**) Blurry images simulated by CODE V; (**b**) The restoring results; (**c**) The ground-truth.

Based on the CODE V simulation data set, we train our network, and obtain 34 M parameters after 1000 epochs of training, and finally get blurry removal results in test-dataset, part of them are shown in Figure 5.

Furthermore, we validate our proposed method against several existing supervised methods which are applicable in the current field. The comparison methods include: the non-uniform aberration deblurring method [37], Unet-based restoring method [38], FOV-GAN restoring method [6], DeblurGAN-v2 restoring method [20]. We do not compare with previous simple lens restoring methods [4,5] owing to the complex calibration process. We choose a blind non-uniform aberration deblurring method as the representative of the optimization-based methods [37]. Other restoring methods are based on deep network, which can achieve end-to-end image restoration.

**Figure 6:**
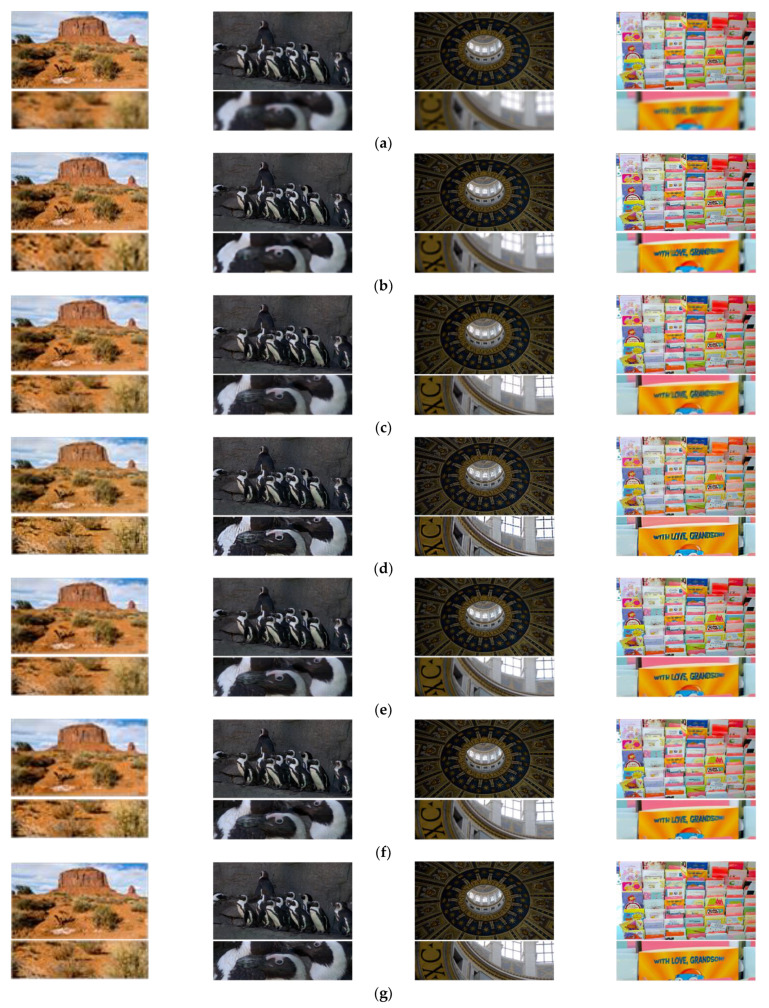
The results comparison using different restoring methods based on simulation dataset: (**a**) Original blurry images; (**b**) Non-uniform aberration deblurring results; (**c**) Unet restoring results; (**d**) FOV-GAN restoring results; (**e**) DeblurGAN-v2 restoring results; (**f**) Our proposed RRG-GAN restoring results; (**g**) The ground truth.

The running environment of this work is: Intel (R) core (TM) i7-6700 CPU @ 3.40GHz, 32GB system memory, NVIDIA GeForce RTX 2080 Ti with 11,264 Mb GPU memory. The training parameters are set according to the original paper, and the training epoch are all set to 1000. The recovery results obtained by the above methods are shown in Figure 6.

Figure 6b is the restoring results of optimization-based theory. Although the estimation of blur kernel has been iterated for many times, it is still not accurate, and thus cannot achieve good restoration of degraded images. In addition, the running time is more than 20 min on one test image with 1920 × 1080 resolution. Other optimization-based methods that can be used in this computational imaging restoration, such as [39,40], the restoring results and running time are similar to this method. Compared with the traditional methods, the restoring methods based on deep learning have a greater improvement in the restoration effect and efficiency. Figure 6c shows the restoration results of Unet, and Figure 6e shows the restoration results of Deblur-GAN-v2. These methods are not as good as our proposed method in the restoration of weeds, text and other complex details. Figure 6d shows the restoration results implemented by FOV-GAN network, which produce some ringing effect in the complex detail texture. The proposed RRG-GAN network achieves better results than other deep-learning-based methods in the restoration of complex detail texture. The restored image edge is clear and natural, and there is no obvious ringing effect, which is more in line with human subjective visual effect. This is the role played by the fusion of attention module and feature module in a generative network.

Since the images in the test dataset are obtained by CODE V simulation, there are true values that can be used for reference. Several objective evaluation indexes can be used to evaluate the above algorithms: Structural SIMilarity (SSIM) [34], Peak Signal to Noise Ratio (PSNR), Root Mean Square Error (RMSE) [41], and Spectral Angle Mapper (SAM) [42].

The quantitative comparisons of restoring performance are shown in Table 1. As can be seen from Table 1, the objective evaluation index obtained by the RRG-GAN network method described in this section is superior to other recovery methods.

The running time and the parameter-size of the above methods are summarized in Table 2. Through the comparison, we find that the running time of deep learning methods is greatly better than the traditional optimization-based method, but worse than other deep-learning-based methods. Compared with other deep-learning-based methods, our network structure is more complex, but the recovery effect is better, as shown in Figure 6. Furthermore, the parameter size of our method is much smaller than other deep learning methods, which is determined by the recursive structure of the network.

### 4.3. Apllication in Real Scene

#### 4.3.1. The Reason of Constructing Manual Registering Dataset

**Figure 7:**
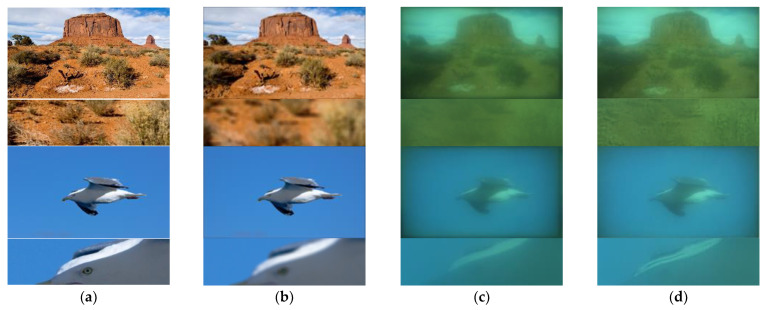
Network restoring results based on simulation dataset fails in real scene test: (**a**) Original sharp images; (**b**) Blurry images simulated by CODE V; (**c**) The real blurry images directly captured by simple lens imaging system; (**d**) The restoring results from (**c**) using the parameters trained by CODE V simulation data set.

Simulation evaluation is an ideal test scenario, and its significance is to provide a validation benchmark for algorithm analysis. However, when the single-convex-lens is actually assembled for imaging, the actual imaging effect is different from CODE V’s simulation, as shown in Figure 7.

From Figure 7, the results show that the network parameters trained by the simulation test set are not effective when deployed in the actual scene. The main reason for this problem is that the data distribution of the simulation dataset is inconsistent with the real scene. In addition to the conventional image blur caused by optical aberration, there are two additional degradation factors, color shift and bright background interference, which are not simulated in CODE V software.

The color shift is caused by the white balance problem of CMOS camera. The bright background interference is caused by straylight. The single-convex-lens imaging system has no special aperture, and the gland ring plays the role of aperture, which makes the straylight easier to produce and has a direct impact on the image formation. Straylight will produce bright background, and further reduces the modulation transfer function (MTF) of the optical system. These two kinds of additional interferences cause the data distribution difference between the simulation dataset and the actual scene, we further need to reconstruct a dataset for the actual scene.

#### 4.3.2. The Constructure of Manual Registering Datasets

**Figure 8:**
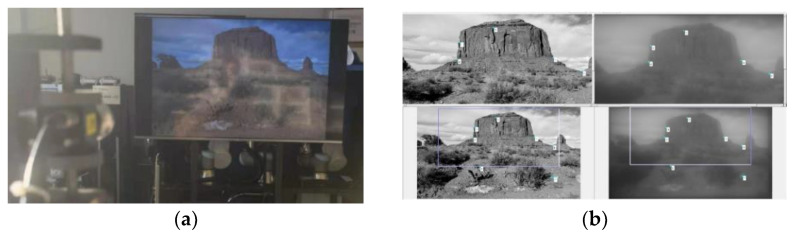
The key procedure constructing manual registering database; (**a**) The display-capture lab setup; (**b**) The interactive selection of artificial feature points in MATLAB software.

Similar to Peng [6], we built a display-capture lab setup as shown in Figure 8a. We display the 200 images in the dataset on an LCD device (52-Inch) and collect them through our single-convex-lens imaging system. The LCD device is placed 8 m away from the camera, and the intrinsic discretization of the LCD screen can be ignored at this distant. There is a large position error due to the mismatch between the original image and the captured image. Therefore, we specially write a MATLAB script program for interactive selection of artificial feature points to register two images, as shown in Figure 8b.

In the manual calibration shown in the Figure 8b, after 10 feature point pairs are selected manually for each image pair, the affine transformation parameters can be calculated by using the least square method. It is found that a set of fine calibrated affine parameters can be applied to all images due to the relatively static acquisition environment, and there is no need to calibrate each image. Therefore, we obtained a dataset contains 200 “true value-picking up” registered samples.

#### 4.3.3. Algorithm Evaluation Based on Manual Registering Datasets

**Figure 9:**
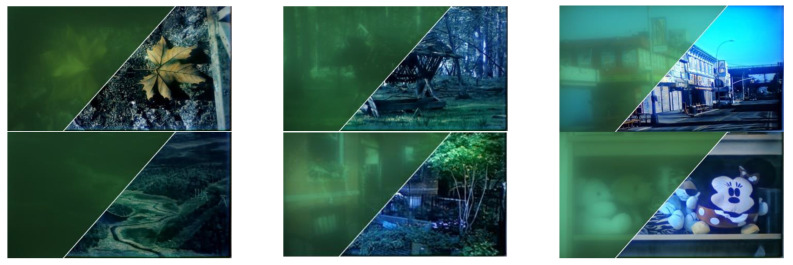
RRG-GAN restoring results based on manual dataset.

Again, we select 128 groups as training datasets, 36 groups as validation datasets and 36 groups as test datasets. After 1000 epochs of training process, the restoring images are shown in Figure 9. From the effect of image restoration, we can see that our method can not only eliminate the optical blur, but also correct the color deviation and bright background interference.

**Figure 10:**
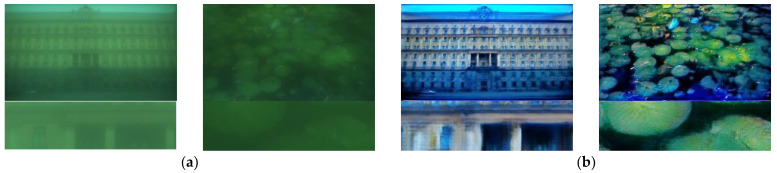

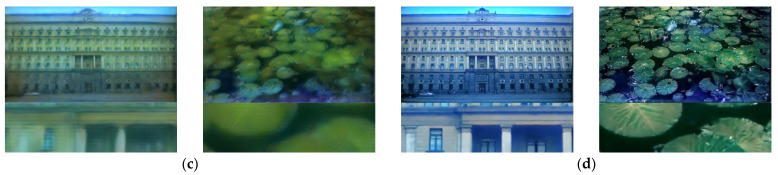
The results comparison using different deep-learning methods based on manual dataset: (**a**) Original blurry images; (**b**) FOV-GAN restoring results; (**c**) DeblurGAN-v2 restoring results; (**d**) Our proposed RRG-GAN restoring results.

Under the same experimental conditions, we test the effect of our proposed method and the other two deep learning methods on the manual registering dataset, as shown in the Figure 10. From the figure, we can see that our method is superior to the other two methods.

**Figure 11:**
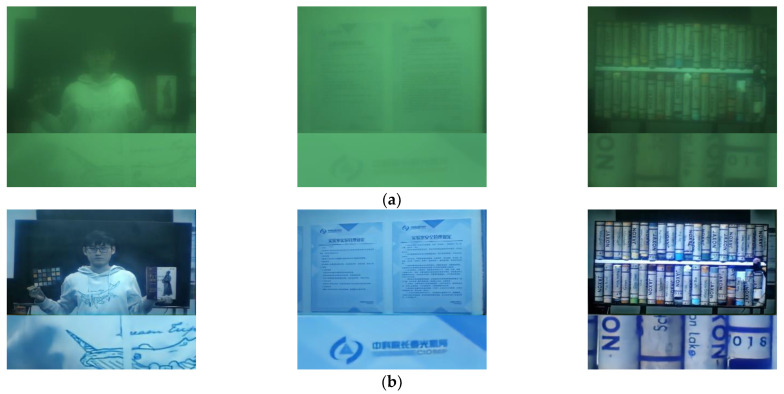
The restoring results in real scenes: (**a**) Images directly captured by single-convex-lens imaging system; (**b**) The restoring images processed by our restoring method.

Furthermore, we apply our restoring method to real scenes, the results are as shown in the Figure 11. Through the actual imaging experiments, it can be seen that after adopting the manual registering dataset training, our proposed storing method can well eliminate three typical image degradation effects of single-convex-lens, including the texture blurry caused by optical aberrations, the color shift caused by the CMOS camera, and the contrast reduction caused by the straylight.

### 4.4. Imaging Effect Comparison with SLR Camera Lens

**Figure 12:**
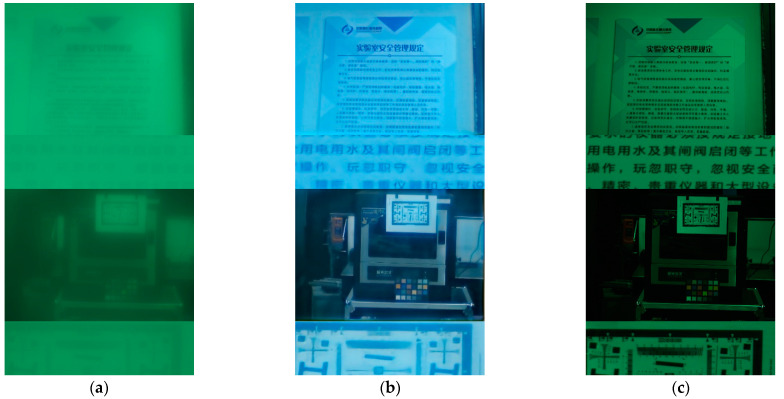
Imaging effect comparison with SLR camera lens: (**a**) Images directly captured by single-convex-lens imaging system; (**b**) The restoring images processed by our restoring method; (**c**) Images directly captured by conventional SLR camera lens.

In this section, we compare the restoring effect obtained by single-convex-lens imaging system with the direct imaging effect using conventional SLR camera lens. We stabilize the camera and capture the scene by these two lenses separately. The imaging effect are shown in Figure 12.

From Figure 12, we can see that the experimental images processed by our restoring method have a good restoration effect for the edge information of the natural scene, and the restoring effect is close to the direct imaging effect using conventional SLR Lens. However, for the special texture such as text, fringe pattern, due to the lack of relevant sample set in the database, it cannot have a good detail recovery effect, which will be improved in the following work.

## 5. Discussion

### 5.1. Improvement Analysis

#### 5.1.1. Efficiency Improvement over Traditional Methods

Due to the indirect imaging mechanism, computational imaging is also faced with the problems of “accurate description of forward modulation transformation model” and “robustness of reverse reconstruction algorithm”. With such problems, especially the particularity of single-convex-lens’s ill conditioned problem, traditional methods need to utilize many iterations to get the accurate description of forward modulation transformation model, while requiring other iterations to ensure the robustness of reverse reconstruction algorithm. However, the deep-learning-based network can avoid the above problems in an end-to-end way, and efficiently restore the latent images from the blurry source data.

#### 5.1.2. Effect Improvement over Deep Learning Methods

Compared with other restoring networks like U-net [38], our proposed RRG-GAN network can achieve better recovery effect. This is determined by the adversarial training process of the generator and discriminator. During training, the generator is trained to produce latent images which can “fool” the discriminator network, and the discriminator is trained to better distinguish the images captured by the single-convex-lens and the latent images generated from the generator.

Our RRG-GAN network can achieve better detail recovery effect than other GAN networks like Deblur-GAN [20]. This is due to the use of multi-scale dual attention blocks module, which can simulate the process of human eyes searching for important information in the visual scene for centralized analysis while ignoring irrelevant information in the scene. The selective kernel networks are used combined with the dual attention blocks. They can effectively learn to fuse important features in multi-scale. The residual resizing networks provide additional network parameters for learning in the inter-scale up-down-sampling operation, so as to further improve the performance of the whole network.

### 5.2. Limitations and Deficiencies

Like most deep learning methods, the data-driven-based methods require the consistency of the distribution of training data and actual data. However, the mechanism of computational imaging system determines that it is difficult to obtain the ideal training data which is consistent with the actual data. Through the experimental results in Section 2.3, we find that although the color restoration is greatly improved compared with the original image, it still retains a certain color offset, which is caused by the background light of LCD display.

Furthermore, small errors are inevitable when we manually register the dataset. Although our RRG-GAN network can overcome this small error, it will still affect the performance of network. Therefore, how to design the training dataset more reasonably is also one of our further works.

### 5.3. Further Work

Aberration is the core problem of optical design. The aberration correction method based on computational imaging has an important significance in other optical systems. Especially in some special optical imaging scenes, under the premise of limited cost and limited optical lens size, the traditional optical design method is difficult to meet the design needs. In this case, it is a very feasible scheme to allow optical designers to reduce optical index and keep proper aberrations, and deliver aberration correction work to calculation and restoration algorithm. Our following work will try to apply our restoring method to complex optical systems, such as reflective optical systems, to achieve the joint optimization of optical design and restoration algorithm design, so as to achieve a higher index of computational imaging system design.

## 6. Conclusions

In this paper, we propose a novel RRG-GAN network to restore the latent images from blurry source data captured by a single-convex-lens imaging system in an end-to-end way. We restructure the generator network, which includes a dual attention module, selective kernel network module, and a residual resizing module. These improvements make RRG-GAN more suitable for the non-uniform deblurring task. We collect sharp/aberration-degraded datasets by CODE V simulation and manual registering. Relevant experimental comparisons and actual tests verify the effectiveness of our proposed method.

## Figures and Tables

**Table 1:** Quantitative comparisons of restoring performance.

	SSIM	PSNR	RMSE	SAM
Original	0.6278	24.4386	16.3857	4.5950
Multiscale	0.6859	25.0048	15.1276	4.7078
Unet	0.7922	28.3427	10.6450	4.1911
Fov-GAN	0.7486	25.2653	14.4563	5.6238
Deblur-GAN	0.7843	28.0252	10.9077	4.5637
RRG-GAN	0.8650	30.4102	8.3224	3.7855

**Table 2:** Statistics of running time and network parameter size.

	MultiScale	Unet	FovGAN	DeblurGAN-V2	RRG-GAN
Runtime	25 min	0.489 s	0.714 s	2.677 s	2.738 s
Para size	/	363.701 MB	83.852 MB	238.982 MB	31.494 MB

## Data Availability

The data presented in this study are openly available in Github at (https://github.com/wuzeping1893/RRG-GAN-single-convex-lens).

## Abbreviations

RRG	Recursive Residual Groups
GAN	Generative Adversarial Networks
CNN	Convolutional Neural Networks
RNN	Recurrent Neural Networks
CMOS	Complementary Metal Oxide Semiconductor
PSF	Point Spread Function
MRB	Multi-scale Residual Block
MAP	Maximum A Posteriori Approach
VEM	Variational Expectation Maximization
MTF	Modulation Transfer Function
LCD	Liquid Crystal Display
SLR	Single-convex-lens Reflex
